# Trends in Disparities and Transitions of Treatment in Patients With Early Breast Cancer in China and the US, 2011 to 2021

**DOI:** 10.1001/jamanetworkopen.2023.21388

**Published:** 2023-06-30

**Authors:** Jianbin Li, Jifang Zhou, Haibo Wang, Zhenzhen Liu, Zhimin Fan, Yinhua Liu, Cuizhi Geng, Yue Xiao, Zefei Jiang

**Affiliations:** 1 Senior Department of Oncology, The Fifth Medical Center of Chinese People’s Liberation Army General Hospital, Beijing, China; 2 Department of Medical Molecular Biology, Institute of Biotechnology, Academy of Military Medical Sciences, Beijing, China; 3 Department of Public Administration, China Pharmaceutical University, Jiangning Campus, Nanjing, China; 4 Breast Cancer Center, Affiliated Hospital of Qingdao University, Qingdao, China; 5 Department of Breast Disease, Affiliated Cancer Hospital of Zhengzhou University, Henan Cancer Hospital, Zhengzhou, China; 6 Department of Breast Surgery, General Surgery Center, First Hospital of Jilin University, Changchun, China; 7 Department of Breast Surgery, Peking University First Hospital, Beijing, China; 8 Breast Cancer Center, Fourth Hospital of Hebei Medical University, Shijiazhuang, China

## Abstract

**Importance:**

Breast cancer treatment has profoundly improved in China recently. However, trends in disparities and transitions of treatment in early-stage cancer between China and the US are not well known.

**Objective:**

To identify changes for patients with early breast cancer by using large databases from China and the US.

**Design, Setting, and Participants:**

This multicenter cross-sectional study used the Chinese Society of Clinical Oncology Breast Cancer (CSCO BC) database from hospitals in 13 provinces in China and the Flatiron Health (hereinafter referred to as Flatiron) database from more than 280 community oncology clinics in the US. Patients with stage I to III breast cancer diagnosed from January 1, 2011, to December 31, 2021, were included. Data were analyzed from June 10 to December 1, 2022.

**Main Outcomes and Measures:**

The distribution of age, clinical stage, and cancer subtypes at diagnosis were examined overall and by year. The mean annual percent change (MAPC) from 2011 to 2021 in systemic therapy and surgery was also analyzed.

**Results:**

A total of 57 720 patients with early breast cancer were screened from the CSCO BC (n = 45 970) and Flatiron (n = 11 750) databases. The median age at diagnosis in China among the 41 449 patients included in the age analysis was 47 (IQR, 40-56) years; in the US, the median age was 64 (IQR, 54-73) years. Among patients with clinical stage data in the CSCO BC (n = 22 794) and Flatiron (n = 4413) databases, the proportion of stage I cancer was 7250 (31.8%) vs 2409 (54.6%); stage II cancer, 10 043 (44.1%) vs 1481 (33.6%); and stage III cancer, 5501 (24.1%) vs 523 (11.9%). The proportion of hormone receptor–positive cancer in China was 69.8%, lower than that in the US (87.5%). For patients with *ERBB2* (formerly *HER2* or *HER2/neu*)-positive cancer, the proportion in China (30.2%) was higher than that in the US (15.6%). For neoadjuvant therapy, the annual rate increased from 247 of 1553 (15.9%) to 200 of 790 (25.3%) in China, with an MAPC of −4.4% (95% CI, −50.6% to 85.0%; *P* = .89). For patients with *ERBB2*-positive cancer, the proportion treated with trastuzumab in early-stage cancer in China increased significantly, with an MAPC of 22.1% (95% CI, 17.4%-26.9%; *P* < .001), and overtook that in the Flatiron database since 2017 (1684 [68.5%] vs 550 [62.5%]; *P* < .001).

**Conclusions and Relevance:**

The findings of this cross-sectional study suggest that disparities in treatment of early breast cancer narrowed between China and the US during the study period. The rapid growth of trastuzumab treatment in China was suggestive of differential access to targeted *ERBB2* therapy.

## Key Points

**Question:**

What are the trends and disparities of treatment among women with early breast cancer in China and the US?

**Findings:**

In this cross-sectional study of 57 720 patients with early breast cancer diagnosed from 2011 to 2021, patients in China were younger and had more late-stage and aggressive cancer subtypes. Of these, 69% of patients with *ERBB2* (formerly *HER2* or *HER2/neu*)-positive cancer received trastuzumab-based therapy after 2017 in China compared with 62% of patients in the US during the same period.

**Meaning:**

These findings suggest that disparities in early breast cancer treatment between patients in China and patients in the US narrowed gradually from 2011 to 2021.

## Teaser

This cross-sectional study compares trends in diagnosis and treatment patterns among women with early breast cancer in China and the US.

## Introduction

Breast cancer incidence has been slowly increasing by about 0.5% per year since the mid-2000s.^1^ Its mortality has almost halved due to the wide application of mammography screening^2^ and improvements in treatment.^3^ The expansion of medical care was also associated with a reduced incidence of late-stage breast cancer,^4^ which contributed to further declines in mortality and incidence of breast cancer.

China has an increasing burden and mortality of breast cancer,^5^ and disparities in incidence, stage at diagnosis,^6^ and mortality^7^ between patients from China and the US persist. A series of actions has been taken in China to relieve its cancer burden and to bridge these gaps, with acceleration of effective and affordable drug delivery,^8^ promotion of standardized guidelines,^9^ and emergence of new landscapes for clinical trials.^10^ These comprehensive strategies have offered intensive guidance to facilitate cancer treatment that appears to have survival superiorities over existing treatments on preliminary evidence.^11^

However, the transitions and disparities in early breast cancer between China and the US are still controversial. Data on the characteristics of early breast cancer and how they have changed over time in the 2 countries are scarce. The Chinese Society of Clinical Oncology Breast Cancer (CSCO BC) database established in 2017 has made the deep mining of longitudinal data possible. Meanwhile, the Flatiron Health (hereinafter referred to as Flatiron) longitudinal database contains patient demographic characteristics and clinical and surgery-related management. Given the importance, the CSCO BC Committee initiated a clinical program through 2 large databases (CSCO BC-US01). To fill the evidence gap, the primary aim of our study was to identify the differences in changes in diagnosis and treatment patterns for early breast cancer. The second aim was to compare patients from both databases during the same period. The results will inform disparities in cancer diagnosis and treatment between China and the US.

## Methods

This cross-sectional study was conducted in accordance with the principles of Good Clinical Practice and the Declaration of Helsinki. Use of the Flatiron database was approved by the China Pharmaceutical University Institutional Review Board, which waived the need for informed consent. Use of the CSCO BC database was approved by the Ethics Board of the Affiliated Hospital of Qingdao University. Oral informed consent was obtained from CSCO BC study participants. The study followed the Strengthening the Reporting of Observational Studies in Epidemiology (STROBE) reporting guideline.

### Study Design and Participants

This study used deidentified patient data from the CSCO BC database and Flatiron analytic database. The hospital-based CSCO BC database is initiated by the CSCO BC Committee to collect data from multiple centers, including more than 100 000 patients from hospitals in 13 provinces with detailed, high-quality breast cancer data on stage at diagnosis, surgery, systemic therapy, and time from diagnosis to metastasis. The Flatiron database is a clinic-based, longitudinal, deidentified electronic health record–derived database that includes structured and unstructured data from 280 US cancer clinics with approximately 800 sites of care curated via technology-enabled abstraction.^12^ Most of the patients in the Flatiron database were treated at community oncology clinics not affiliated with teaching institutions.

Patients were included in the analysis if they were women 18 years or older; were diagnosed with invasive breast cancer; had received at least 1 therapy such as neoadjuvant therapy, surgery, or adjuvant therapy; and had a minimum potential follow-up for at least 3 months from the index date to the study cutoff date of December 31, 2021. Patients were excluded if they were diagnosed with de novo stage IV breast cancer or ductal or lobular carcinoma in situ or were diagnosed before January 1, 2011.

### Quality Control

Epidemiological and demographic data and clinical treatment were extracted from the databases. Missing data were inevitable, especially in the early years. Excluding all these data would produce a spurious treatment distribution, which would have led us to underestimate the real changes of cancer treatment. To avoid this bias, internal logic was used to fill the missing data. For TNM stage, clinical stage at diagnosis was prioritized. If data on clinical stage were missing, pathological stage data were used instead for patients who received surgery as their initial therapy. Cases were identified as unknown and were excluded under specific analysis conditions if information was missing. Information about race and ethnicity were not included due to the absence of these data in the CSCO BC database.

### Statistical Analysis

Data were analyzed from June 10 to December 1, 2022. The distribution of age, stage, and molecular classification at diagnosis was examined overall and by years. We also analyzed the 10-year trends from 2011 to 2021 in systemic therapy and surgery. For patients with *ERBB2* (formerly *HER2* or *HER2/neu*)-positive tumors, trastuzumab-based therapy, including trastuzumab biosimilars, was also analyzed over the decade.

The annual increase in rates was calculated for each indicator and defined by the initial date of their treatment and diagnosis. Statistical modeling considered the chronological time under analysis as an independent variable. The annual percent change (APC), mean APC (MAPC), and their 95% CIs were calculated by using joinpoint regression.^13^ The trend was considered significant when the model resulted in *P* < .05. If the accurate data of diagnosis or treatment was missing, the most relevant therapeutic date was regarded as a substitution.

For descriptive analyses, number (percentage) was used for qualitative variables. We used a log-rank test to compare the difference between different databases after excluding these missing data. A 2-sided α < .05 was considered statistically significant. Statistical analysis was performed using SAS, version 9.4 (SAS Institute Inc). Data from the 2 databases were separately analyzed by 2 different statisticians (J.L. and J.Z.) with the same standard. Figures were drawn using GraphPad Prism, version 8 (GraphPad Software Inc). Joinpoint was calculated and drawn using the Joinpoint Regression Program, version 5.0.1 (National Cancer Institute).

## Results

After excluding 9472 cases diagnosed before 2011, a total of 57 720 patients with early breast cancer were included, with 45 970 patients from the CSCO BC database and 11 750 patients from the Flatiron database. All of them were women. Most patients from the CSCO BC database were from the eastern part of the country (eFigure 1 in Supplement 1). The demographic and clinical characteristics of the study population overall and by database are shown in Table 1.

**Table 1.** Characteristics of Patients With Early Breast Cancer From 2 Databases^a^

Characteristic	Database	
	CSCO BC (n = 45 970)	Flatiron Health (n = 11 750)
Age, median (IQR), y	47 (40-56)	64 (54-73)
Diagnosis time		
2011-2016	33 427 (72.7)	5684 (48.4)
2017-2021	12 543 (27.3)	6066 (51.6)
Menstrual status		
Premenopausal	24 226 (52.7)	2077 (19.7)
Postmenopausal	21 744 (47.3)	8492 (80.3)
Clinical stage		
I	7250 (31.8)	2409 (54.6)
II	10 043 (44.1)	1481 (33.6)
III	5501 (24.1)	523 (11.9)
Phenotypes		
HR positive–*ERBB2* negative	15 079 (53.9)	8708 (75.5)
HR positive–*ERBB2* positive	4439 (15.9)	1383 (12.0)
HR negative–*ERBB2* positive	4007 (14.3)	416 (3.6)
TNBC	4439 (15.9)	1025 (8.9)
Neoadjuvant therapy	10 642 (23.1)	1804 (15.4)
Surgery	41 511 (90.3)	11 316 (96.3)
Adjuvant therapy	27 122 (59.0)	8664 (73.7)

Abbreviation: CSCO BC, Chinese Society of Clinical Oncology Breast Cancer; *ERBB2*, formerly *HER2* or *HER2/neu*; HR, hormone receptor; TNBC, triple-negative breast cancer.

^a^ Unless otherwise indicated, data are expressed as No. (%) of patients. Patients with missing data were excluded when calculating the proportions.

A total of 41 449 patients in the CSCO BC database were included in the age analysis. The median age at cancer diagnosis in China was 47 (IQR 40-56) years. A total of 14 549 women (35.1%) were diagnosed at 40 to 49 years of age, and 10 031 (24.2%) were diagnosed at 50 to 59 years of age (eFigure 2 in Supplement 1). Only 1658 patients (4.0%) were older than 69 years. Among the 11 750 patients in the Flatiron database, the median age at cancer diagnosis was 64 (IQR, 54-73) years, with 3419 patients (29.1%) diagnosed at 60 to 69 years of age and 3979 patients (33.9%) older than 69 years.

A total of 22 794 patients in the CSCO BC database and 4413 patients in the Flatiron database were included with detailed clinical stage and accurate date. Among patients in the CSCO BC database, 7250 (31.8%) had stage I cancer, 10 043 (44.1%) had stage II cancer, and 5501 (24.1%) had stage III cancer, with a statistical difference when compared with patients in the Flatiron database (2409 [54.6%] with stage I, 1481 [33.6%] with stage II, and 523 [11.9%] with stage III) (eFigure 3 in Supplement 1).

Considering phenotypes, a total of 27 964 patients in the CSCO BC database and 11 532 in the Flatiron database were analyzed. The proportion of hormone receptor (HR)–positive cancer in China was 69.8% compared with 87.5% in the US. For patients with *ERBB2*-positive cancer, the proportion in China (30.2%) was higher than that in the US (15.6%). Patients with HR-positive–*ERBB2*-negative cancer constituted 53.9% of the CSCO BC and 75.5% of the Flatiron databases; those with HR-positive–*ERBB2*-positive cancer, 15.9% of the CSCO BC and 12.0% of the Flatiron databases; those with HR-negative–*ERBB2*-positive cancer, 14.3% of the CSCO BC and 3.6% of the Flatiron databases; and those with triple-negative cancer, 15.9% of the CSCO BC and 8.9% of the Flatiron databases. Clinical stages and phenotypes at diagnosis varied over the decades in both databases (eFigure 4 in Supplement 1).

**Figure 1.**
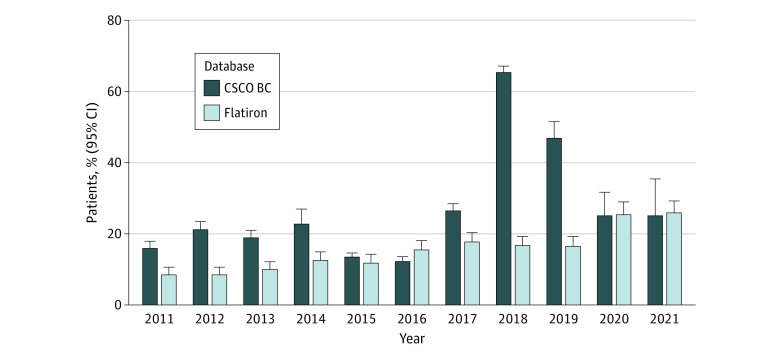
Proportions of Neoadjuvant Therapy Participants included patients with early breast cancer in the Chinese Society of Clinical Oncology Breast Cancer (CSCO BC) database and Flatiron Health (Flatiron) database from January 1, 2011, to December 31, 2021. Error bars represent 95% CIs of estimated margins.

A total of 10 642 patients in the CSCO BC database (23.1%) received neoadjuvant therapy. The annual rate increased from 247 of 1553 (15.9%) in 2011 to 200 of 790 (25.3%) in 2021, with an MAPC of −4.4% (95% CI, −50.6% to 85.0%; *P* = .89) (Figure 1 and eFigure 5 in Supplement 1). There was a significant increase of neoadjuvant therapy from 2016 to 2019 (APC = 103.6% [95% CI, 13.7% to 264.6%]; *P* = .03). The annual rate in the US increased from 81 of 957 (8.5%) in 2011 to 232 of 897 (25.9%) in 2021, with an MAPC of 12.1% (95% CI, 9.3%-15.0%; *P* < .001).

**Figure 2.**
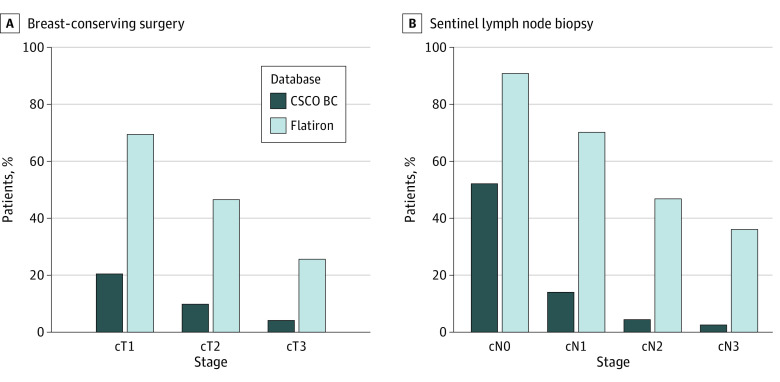
Proportions of Breast-Conserving Surgery and Sentinel Lymph Node Biopsy According to Clinical Stages Patients receiving neoadjuvant therapy were excluded. Breast-conserving surgery was stratified by different clinical T (cT) stages; sentinel lymph node biopsy, by different clinical N (cN) stages. CSCO BC indicates Chinese Society of Clinical Oncology Breast Cancer.

Among patients who had not received neoadjuvant therapy, the conserving surgery rate for patients with clinical T1 (cT1) lesions was 2132 of 10 230 (20.8%) in the CSCO BC database and 1491 of 2141 (69.6%) in the Flatiron database (Figure 2). A significant increase of breast-conserving therapy was observed in the Flatiron database, with an MAPC of 0.9% (95% CI, 0.3%-1.5%; *P* = .01) (eFigures 6 and 7 in Supplement 1). For patients with clinical N0 (cN0) lesions, the percentage of sentinel lymph node biopsy was 6180 of 11 819 (52.3%) in the CSCO BC database and 7652 of 8414 (90.9%) in the Flatiron database. In the CSCO BC database, the biopsy rate for cN0 ranged from 31 of 269 (11.5%) in 2011 to 60 of 80 (75.0%) in 2021, with an MAPC of 20.8% (95% CI, 6.6%-36.8%; *P* = .003) (eFigure 8 in Supplement 1). Despite no significant changes observed (MAPC = 0.1% [95% CI, −0.3% to 0.4%; *P* = .75]) in the Flatiron database, the year 2018 was an inflection point between increasing and decreasing proportions concerning sentinel lymph node biopsy for cN0.

**Figure 3.**
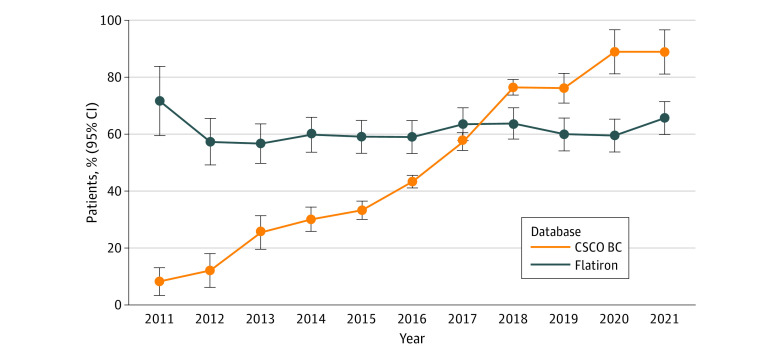
Trends of Annual Proportions of Trastuzumab Received by Patients With *ERBB2*-Positive Cancer Participants included patients with early breast cancer in the Chinese Society of Clinical Oncology Breast Cancer (CSCO BC) and Flatiron Health (Flatiron) databases from January 1, 2011, to December 31, 2021. Error bars represent 95% CIs of estimated margins. Trastuzumab was received in neoadjuvant or adjuvant settings. *ERBB2* indicates formerly *HER2* or *HER2/neu*.

For patients with *ERBB2*-positive cancer in the CSCO BC database, the trastuzumab-based therapy in neoadjuvant or adjuvant settings increased significantly over time from 11 of 128 (8.6%) in 2011 to 56 of 63 (88.9%) in 2021, with an MAPC of 22.1% (95% CI, 17.4%-26.9%; *P* < .001) (Figure 3 and eFigure 9 in Supplement 1). The APC showed a faster increase in the CSCO BC database (29.7% [95% CI, 22.7%-37.1%]; *P* < .001) before 2018. On the contrary, with a median of 13.1 months of follow-up (7.7 months for trastuzumab-based therapy and 14.8 months for non–trastuzumab-based therapy), trastuzumab use in the US decreased from 38 of 53 (71.7%) to 173 of 263 (65.8%), with an MAPC of 0.6% (95% CI, −0.7% to 1.9%; *P* = .33).

Overall, the proportions of patients receiving neoadjuvant therapy, breast-conserving surgery for cT1 lesions, and sentinel lymph node biopsy for cN0 lesions were significantly increased since 2017 (Table 2). In 2011 to 2016, the proportion of trastuzumab administered in the CSCO BC database was significantly lower than that in the Flatiron database (1336 [36.3%] vs 589 [58.6%]; *P* < .001). The use of trastuzumab-based therapy in the CSCO BC database surpassed that in the Flatiron database since 2017 (1684 [68.5%] vs 550 [62.5%]; *P* < .001).

**Table 2.** Changes in Timely Treatment^a^

Therapy by database	Patients		*P* value
	2011-2016	2017-2021	
**Neoadjuvant therapy**			
CSCO BC	5182/33 218 (15.6)	5460/12 494 (43.7)	<.001
Flatiron Health	718/6355 (11.3)	1034/5178 (20.0)	<.001
*P* value	<.001	<.001	NA
**Breast-conserving surgery for clinical T1 lesions**			
CSCO BC	1381/7620 (18.1)	717/2395 (29.9)	<.001
Flatiron Health	2938/4058 (72.4)	2440/3260 (74.8)	.02
*P* value	<.001	<.001	NA
**Sentinel lymph node biopsy for clinical N0 lesions**			
CSCO BC	3149/6745 (46.7)	1520/2188 (69.5)	<.001
Flatiron Health	4046/4448 (91.0)	3500/3785 (92.5)	.01
*P* value	<.001	<.001	NA
**Target therapy for patients with *ERBB2*-positive cancer**			
CSCO BC	1336/3676 (36.3)	1684/2458 (68.5)	<.001
Flatiron Health	589/1005 (58.6)	550/880 (62.5)	.09
*P* value	<.001	<.001	NA

Abbreviations: CSCO BC, Chinese Society of Clinical Oncology Breast Cancer; *ERBB2*, formerly *HER2* or *HER2/neu*; NA, not applicable.

^a^ Data are expressed as No./total No. (%) of patients.

## Discussion

The findings of this cross-sectional study suggest that there were huge disparities in diagnosis and treatment of early breast cancer between China and the US during the study period. We found that patients in China were younger and had a higher proportion of late-stage and aggressive subtypes at diagnosis compared with patients in the US. These disparities may also be associated with the higher proportion of neoadjuvant therapy in China. For those who had not received neoadjuvant therapy, rates of both breast-conserving surgery and sentinel lymph node biopsy were relatively lower in China than in the US. The rate of breast-conserving surgery in China is only one-third lower than that in the US for patients with cT1 lesions. However, the disparities narrowed over the past decade. Especially for patients with *ERBB2*-positive cancer, trastuzumab use increased 10-fold in China and surpassed that in the US since 2017.

The diversity of epidemiology in breast cancer between China and the US has become an international consensus.^14^ On one hand, racial and ethnic differences might contribute to the differences in tumor pathogenesis.^15^ On the other hand, the cost-effectiveness of screening women with both ultrasonography and mammography in China is still uncertain.^16^ This contradiction, as well as insufficient health education and promotion,^17^ may lead to a late visit to a hospital and ultimately a late diagnosed clinical stage. Late stage and aggressive phenotype at diagnosis may account for the elevated cancer burden and mortality in China. Fortunately, younger age may be conducive to alleviate its aggravated survival.^18^ The increasing proportion of HR-positive and stages I and II cancer in China over the decade indicate that the breast cancer profile of China is changing from a that of a developing country to a developed country,^19^ which may also be beneficial to improve the survival outcomes. However, in these 2 databases, we were unable to see the changes of mammographic screening that would influence the stage distribution toward an earlier stage.^20^ Exclusion of patients with ductal or lobular carcinoma in situ, due to the limited data, made it difficult to attribute these epidemiological differences to selection bias or real outcomes. In terms of the database, the distinction in population selection between the 2 databases could also bring about the diversity of epidemiology. A wider range of data collection from clinics may have more representation than that from hospitals. This further explains that more than 80% of the population in the Flatiron database had HR-positive cancer, which is consistent with the characteristics of breast cancer reported by Surveillance, Epidemiology, and End Results^21^ or other population-based surveys.^22^ The gap between China and the US may be narrowed if we choose the Chinese patients from a wide range of clinics instead of cancer centers only.

The subtypes and stages at diagnosis could have a direct association with the selection of neoadjuvant therapy.^23^ Compared with patients with early-stage cancer and moderate subtypes, those diagnosed with late-stage and aggressive subtypes are more likely to receive neoadjuvant treatments and are substantially more likely to have a worse prognosis.^24^ The findings of the present study suggest that China’s overall use of neoadjuvant therapy is much higher than that of the US. In the CSCO BC database, we can find a significant increase in neoadjuvant therapy from 2017 to 2019. The CSCO BC guideline established its first standards in neoadjuvant therapy since 2017. Those with aggressive molecular types and late stage at diagnosis were encouraged to receive neoadjuvant therapy in China. The promotion of guidelines was an important factor affecting the increase in use of neoadjuvant therapy. However, the outbreak of COVID-19 contributed to the suboptimal declines and delays for early breast cancer screening.^25^ Under this circumstance, a decrease can be found in the proportion of neoadjuvant therapy in China.

For patients who had not received neoadjuvant therapy, there were significant differences in breast-conserving surgery for different stages, especially for those with cT1 lesions, and the proportion of patients who choose sentinel lymph node biopsy in China is still trailing that of the US. Notably, progress has been made in China in the promotion of breast conservation and sentinel lymph node biopsy.^26^ In the present study, we observed significant growth in sentinel lymph node biopsy among patients with cN0 lesions.

For patients with *ERBB2*-positive cancer, there was a 10-fold increase in the use of trastuzumab-based therapy for early breast cancer from 2011 to 2021 in China. Several reasons may explain this exponential growth. First, China has experienced both economic and epistemological transitions within the past few decades. With the effort of China’s health reform,^27^ the number of cancer drug approvals has increased sharply since 2017, reaching levels similar to those in the US and the European Union.^28^ Second, in July 2017, trastuzumab was first listed in China’s reimbursement drug lists for *ERBB2*-positive breast cancer. The updated national reimbursement drug lists were implemented to incentivize domestic use of target drugs^29^ and addressed the issue of inaccessibility to target therapy in China. The promotion of standard therapy may also be an important factor affecting the increase of target therapy. By contrast, despite the previous prevalence in the US, the proportion of trastuzumab use decreased over the decade. The use of trastuzumab in the US has been surpassed by that in China since 2017. Data from the Flatiron database were vulnerable to decreased use of trastuzumab due to the age distributions and underlying sources of patients. Previous studies have found that patients 65 years and older are less likely to receive trastuzumab therapy for safety reasons.^30,31^ The actual use of trastuzumab in US populations, particularly in community settings, might be different from that in cancer centers.^31,32^ The short follow-up period and important missing data from clinics may also be responsible for the underestimated proportion. Whatever the reasons, some of the key challenges—such as the inequitable health delivery system and the increasing demand for high-quality and value-based service delivery^33^—remain to be faced, both in China and the US, for the wide application of trastuzumab.

### Limitations

This study has some limitations. First, the quality of the data attributed to underlying sources and inclusion criteria of patients varied. Data in the CSCO BC database were collected from cancer centers, while the Flatiron database was clinic based. This means the disparities between 2 countries might be different, especially in trastuzumab use, if patients were selected using the same criteria. Second, it was a challenge to include all patients with fully accurate information, since considerable data are missing, especially in the earlier years of the study period. To avoid this bias, we carefully excluded those patients with missing data during specific analyses. Third, the differences in patients from different regions were not evaluated; the proportions may be different when compared with clinical settings.

## Conclusions

The findings of this cross-sectional study of patients with early breast cancer suggest that patients in China were younger and were more likely to have late-stage and aggressive subtypes compared with patients in the US. The gaps in breast-conserving surgery for cT1 lesions and sentinel lymph node biopsy for cN0 lesions were large but narrowing. The rapid growth of trastuzumab use in China was suggestive of differential access to targeted *ERBB2* therapy. More investigations, especially population-based studies, are necessary to support our findings.

## Notes

Supplement 1. **eFigure 1.** Distributions of Patients From 2 Databases **eFigure 2.** Age Distributions **eFigure 3.** Annual Changes of Clinical Stages at Diagnosis **eFigure 4.** Annual Changes of Phenotypes at Diagnosis **eFigure 5.** Trends in Neoadjuvant Therapy **eFigure 6.** Annual Changes of Surgery **eFigure 7.** Trends in Breast-Conserving Surgery for Patients With Clinical T1 Lesions **eFigure 8.** Trends in Sentinel Lymph Node Biopsy for Patients With Clinical N0 Lesions **eFigure 9.** Trends in Target Therapy for Patients With *ERBB2*-Positive Cancer

Supplement 2. **Nonauthor Collaborators.** Chinese Society of Clinical Oncology Breast Cancer Committee Database Collaborative Group

Supplement 3.
Data Sharing Statement
