# Supplementary material for: Trends in Disparities and Transitions of Treatment in Patients With Early Breast Cancer in China and the US, 2011 to 2021
Source: JAMA Netw Open. 2023 Jun 30;6(6):e2321388. doi: 10.1001/jamanetworkopen.2023.21388 (PMC10314317; doi:10.1001/jamanetworkopen.2023.21388)
Supplement: Supplement 1. — eFigure 1. Distributions of Patients From 2 Databases eFigure 2. Age Distributions eFigure 3. Annual Changes of Clinical Stages at Diagnosis eFigure 4. Annual Changes of Phenotypes at Diagnosis eFigure 5. Trends in Neoadjuvant Therapy eFigure 6. Annual Changes of Surgery eFigure 7. Trends in Breast-Conserving Surgery for Patients With Clinical T1 Lesions eFigure 8. Trends in Sentinel Lymph Node Biopsy for Patients With Clinical N0 Lesions eFigure 9. Trends in Target Therapy for Patients With ERBB2-Positive Cancer [file jamanetwopen-e2321388-s001.pdf]

Supplementary Online Content

Li J, Zhou J, Wang H, et al; Chinese Society of Clinical Oncology Breast Cancer Committee Database Collaborative Group. Trends in disparities and transitions of treatment in patients with early breast cancer in China and the US, 2011 to 2021. *JAMA Netw Open*. 2023;6(6):e2321388. doi:10.1001/jamanetworkopen.2023.21388

eFigure 1. Distributions of Patients From 2 Databases
eFigure 2. Age Distributions
eFigure 3. Annual Changes of Clinical Stages at Diagnosis
eFigure 4. Annual Changes of Phenotypes at Diagnosis
eFigure 5. Trends in Neoadjuvant Therapy
eFigure 6. Annual Changes of Surgery
eFigure 7. Trends in Breast-Conserving Surgery for Patients With Clinical T1 Lesions
eFigure 8. Trends in Sentinel Lymph Node Biopsy for Patients With Clinical N0 Lesions
eFigure 9. Trends in Target Therapy for Patients With *ERBB2*-Positive Cancer

This supplementary material has been provided by the authors to give readers additional information about their work.

© 2023 Li J et al. *JAMA Network Open*.

eFigure 1. Distributions of Patients From 2 Databases

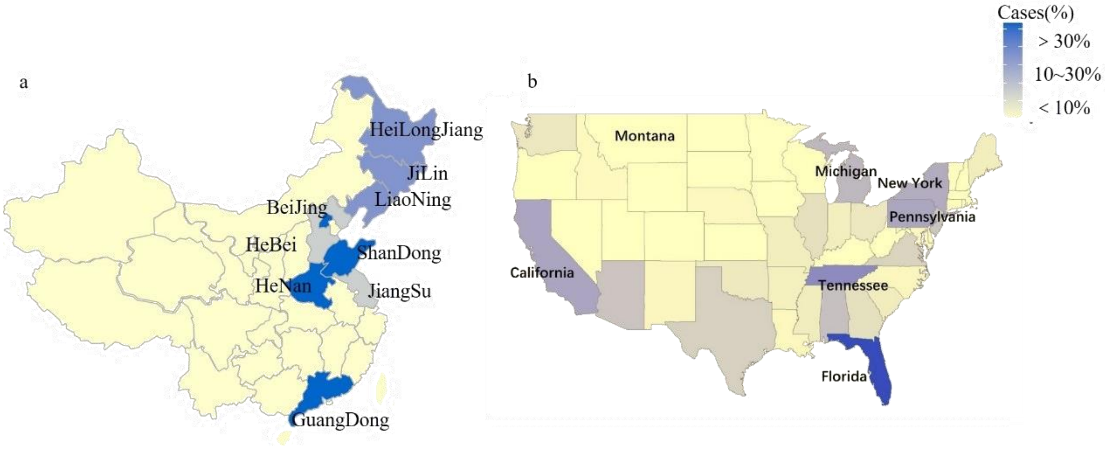

a. patients from CSCO BC database.   b. patients from Flatiron database.

© 2023 Li J et al. *JAMA Network Open*.

eFigure 2. Age Distributions

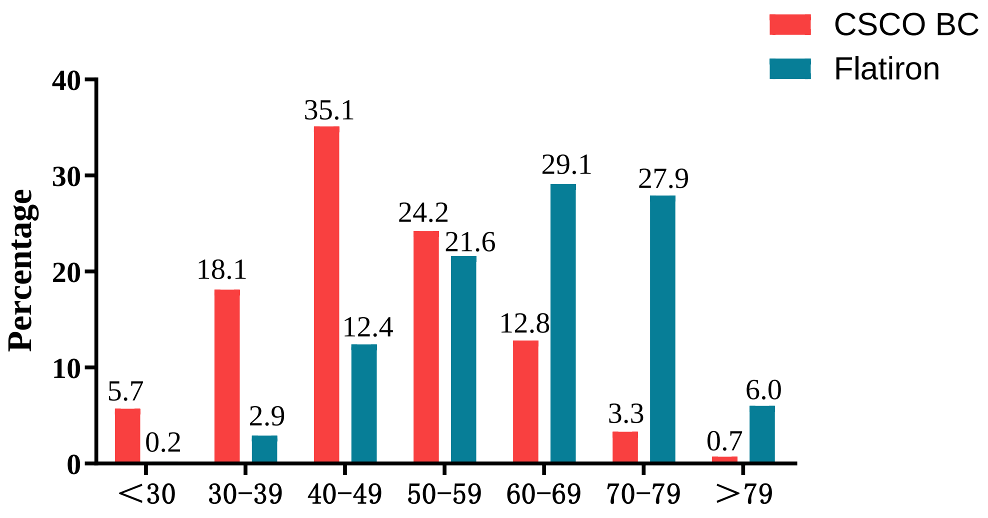

© 2023 Li J et al. *JAMA Network Open*.

eFigure 3. Annual Changes of Clinical Stages at Diagnosis

a, patients from CSCO BC database, b, patients from Flatiron database

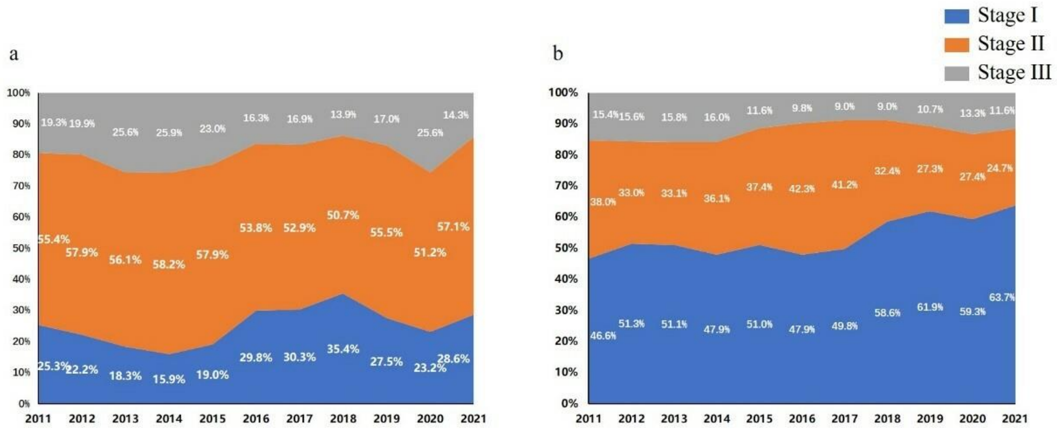

© 2023 Li J et al. *JAMA Network Open*.

eFigure 4. Annual Changes of Phenotypes at Diagnosis

a, patients from CSCO BC database, b, patients from Flatiron database

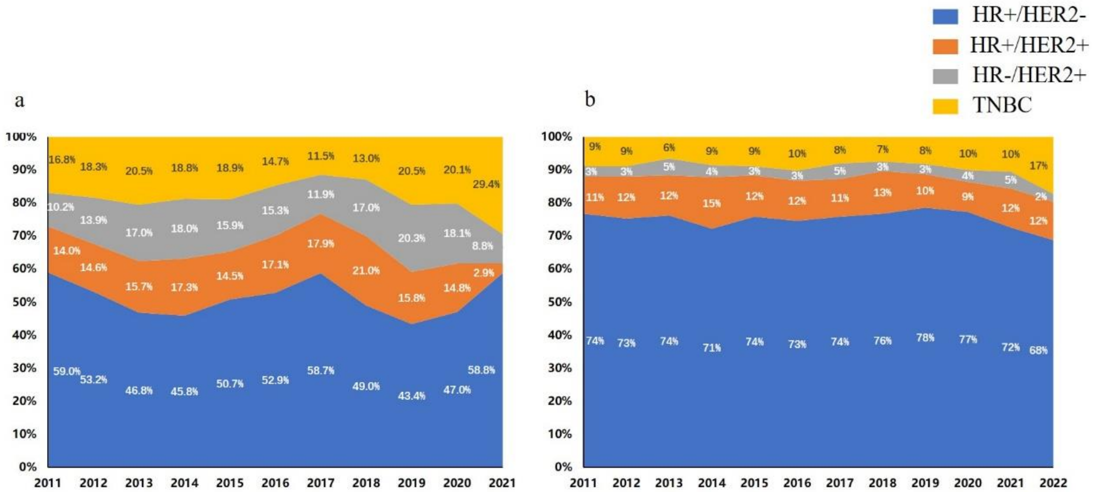

© 2023 Li J et al. *JAMA Network Open*.

eFigure 5. Trends in Neoadjuvant Therapy

a

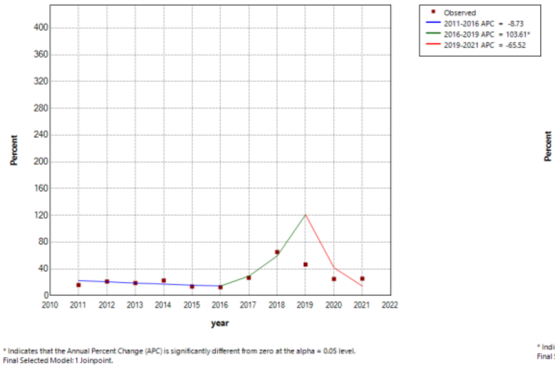

b

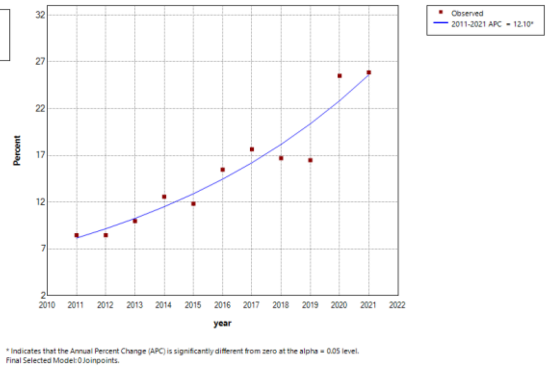

a. joinpoint regression of neoadjuvant therapy for patients from CSCO BC database.

b. joinpoint regression of neoadjuvant therapy for patients from Flatiron database.

In CSCO BC database, AAPC=-4.4% (95%CI: -50.6 to 85.0, p=.89). There were 3 joinpoints: Joinpoint 1(2011-2016), APC= -8.7% (95%CI: -32.2 to 22.9, p=.40). Joinpoint 2(2016-2019), APC=103.6% (95%CI:13.7 to 264.6, p=.03). Joinpoint 3(2019-2021), APC= -65.5% (95%CI: -99.8 to 6385.0, p=.56).

In Flatiron database, there were no joinpoints, AAPC= 12.1% (95%CI:9.3 to 15.0, p < .001).

© 2023 Li J et al. *JAMA Network Open*.

eFigure 6. Annual Changes of Surgery

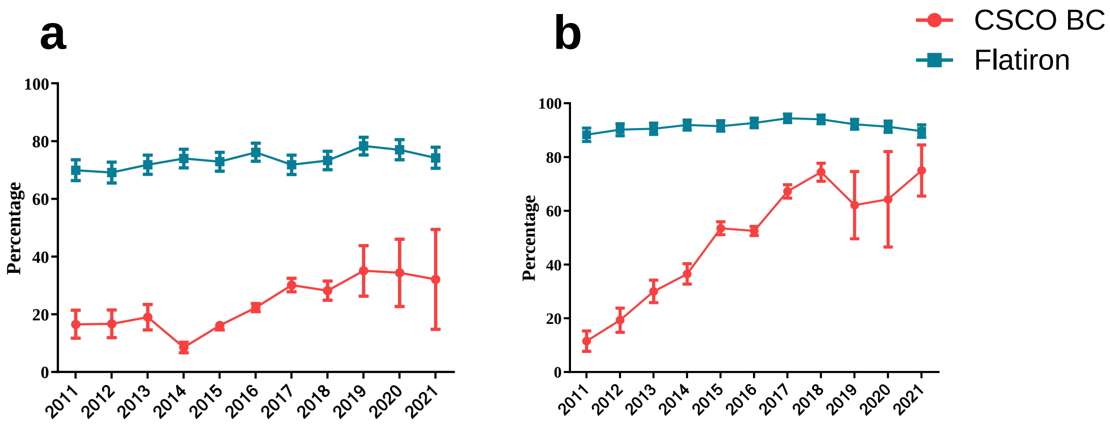

a: breast conserving surgery for cT1; b: sentinel lymph node biopsy cN0

Error bars represent 95% CIs of estimated margins. Patients received neoadjuvant therapy were excluded

© 2023 Li J et al. *JAMA Network Open*.

eFigure 7. Trends in Breast-Conserving Surgery for Patients With Clinical T1 Lesions

a

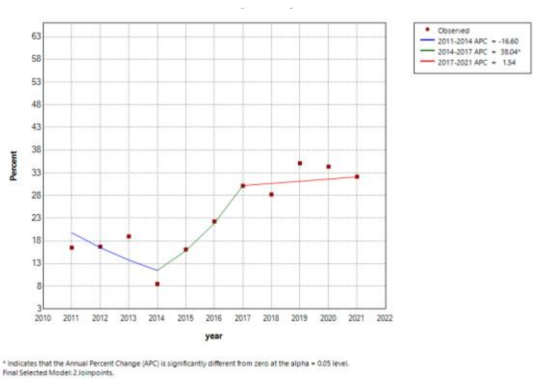

b

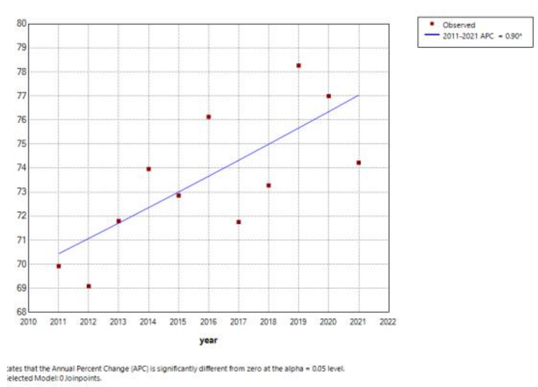

a. joinpoint regression of breast conserving surgery for patients from CSCO BC database.

b. joinpoint regression of breast conserving surgery for patients from Flatiron database.

In CSCO BC database, AAPC=5.0% (95%CI: -0.5 to 10.7, p=.08). There were 3 joinpoints: Joinpoint 1(2011-2014), APC= -16.6% (95%CI: -31.5 to 1.6, p=.06). Joinpoint 2(2014-2017), APC= 38.0% (95%CI:22.9 to 55.0, p=.003). Joinpoint 3(2017-2021), APC= 1.5% (95%CI: -11.1 to16.0, p=.74).

In Flatiron database, there were no joinpoints, AAPC= 0.9% (95%CI:0.26 to 1.54, p=.01).

AAPC: average annual percentage of change, APC: annual percentage of change, for patients received neoadjuvant therapy were excluded

© 2023 Li J et al. *JAMA Network Open*.

eFigure 8. Trends in Sentinel Lymph Node Biopsy for Patients With Clinical N0 Lesions

a

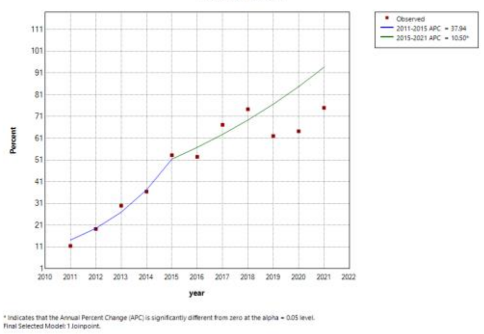

b

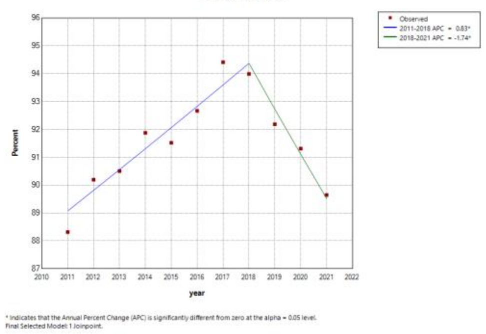

a. joinpoint regression of sentinel lymph node biopsy for patients from CSCO BC database.

b. joinpoint regression of sentinel lymph node biopsy for patients from Flatiron database.

In CSCO BC database, AAPC=20.8% (95%CI: 6.6 to 36.8, p=.003). There were 1 joinpoint: Joinpoint 1(2011-2015), APC=37.9% (95%CI: -4.8 to 99.8, p=.08). Joinpoint 2(2015-2021), APC= 10.5% (95%CI:1.9 to 19.8, p=.02).

In Flatiron database, AAPC=0.05% (95%CI: -0.3 to 0.4, p=.75). There were 1 joinpoint: Joinpoint 1(2011-2018), APC=0.8% (95%CI: 0.5 to 1.1, p < .001). Joinpoint 2(2018-2021), APC=-1.7% (95%CI: -2.8 to -0.7, p=.007).

AAPC: average annual percentage of change, APC: annual percentage of change, for patients received neoadjuvant therapy were excluded

© 2023 Li J et al. *JAMA Network Open*.

eFigure 9. Trends in Target Therapy for Patients With *ERBB2*-Positive Cancer

a

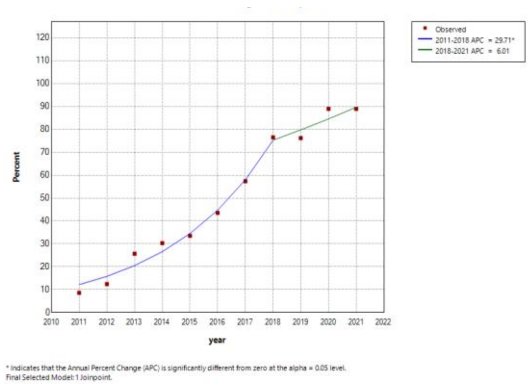

b

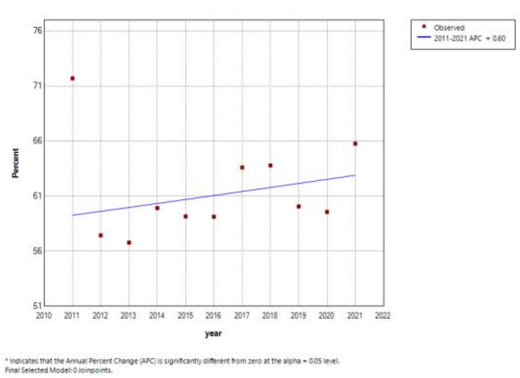

a. joinpoint regression of target therapy for patients from CSCO BC database.

b. joinpoint regression of target therapy for patients from Flatiron database.

In CSCO BC database, AAPC=22.1% (95%CI: 17.4 to 26.9, p < .001). There were 2 joinpoints: Joinpoint 1(2011-2018), APC= 29.7% (95%CI: 22.7 to 37.1, p < .001). Joinpoint 2(2018-2021), APC= 6.0% (95%CI: -3.8 to 16.9, p=.19).

In Flatiron database, AAPC=0.6% (95%CI: -0.7 to 1.9, p=.33). There were no joinpoints.

AAPC: average annual percentage of change, APC: annual percentage of change

© 2023 Li J et al. *JAMA Network Open*.
