# Supplementary material for: Trends in Disparities and Transitions of Treatment in Patients With Early Breast Cancer in China and the US, 2011 to 2021
Source: JAMA Netw Open. 2023 Jun 30;6(6):e2321388. doi: 10.1001/jamanetworkopen.2023.21388 (PMC10314317; doi:10.1001/jamanetworkopen.2023.21388)
Supplement: Supplement 2. — Nonauthor Collaborators. Chinese Society of Clinical Oncology Breast Cancer Committee Database Collaborative Group [file jamanetwopen-e2321388-s002.pdf]

Supplemental Online Content: Nonauthor Collaborators
*First name, last name, and suffix (if applicable) are required and will appear in PubMed.

*Group Name(s): Chinese Society of Clinical Oncology Breast Cancer Committee Database Collaborative Group							
***First Name and Middle Initial(s)**	***Last Name**	***Suffix (eg, Jr, III)**	Academic Degrees	Institution	Location (city, state/province, country)	Role or Contribution, eg, chair, principal investigator	Group (if more than 1 Group listed in the byline) and/or Subgroup (eg, Steering Committee)
Yunjiang	Liu			The Fourth Affiliated Hospital of Hebei Medical University	Shijiazhuang, Hebei, China	principal investigator	
Yuhua	Song			Qingdao University Medical school affiliated hospital	Qingdao, Shandong, China	principal investigator	
Min	Yan			Henan Breast Cancer Center, Affiliated Cancer Hospital of Zhengzhou University & Henan Cancer Hospital	Zhenzhou, Henan, China	principal investigator	
Qianjun	Chen			Guangdong Provincial Hospital of Traditional Chinese Medicine	Guangzhou, Guangdong, China	principal investigator	
Ying	Lin			The First Affiliated Hospital of Sun Yat sen University	Guangzhou, Guangdong, China	principal investigator	
Qiang	Liu			Sun Yat sen University Affiliated Sun Yat sen Memorial Hospital	Guangzhou, Guangdong, China	principal investigator	
Feng	Jin			The First Affiliated Hospital of China Medical University	Shenyang, Liaoning, China	principal investigator	
Yuee	Teng			The First Affiliated Hospital of China Medical University	Shenyang,Liaoning, China	principal investigator	
Peifen	Fu			The First Affiliated Hospital of Zhejiang University	Hangzhou, Zhejiang, China	principal investigator	
Jianguo	Zhang			The Second Hospital affiliated to Harbin Medical University	Harbin, Heilongjiang, China	principal investigator	
Zhigao	Li			Heilongjiang Cancer Hospital	Harbin, Heilongjiang, China	principal investigator	
Yiding	Chen			The Second Affiliated Hospital of Zhejiang University	Hangzhou, Zhejiang, China	principal investigator	

Supplemental Online Content: Nonauthor Collaborators
*First name, last name, and suffix (if applicable) are required and will appear in PubMed.

***First Name and Middle Initial(s)**	***Last Name**	***Suffix (eg, Jr, III)**	Academic Degrees	Institution	Location (city, state/province, country)	Role or Contribution, eg, chair, principal investigator	Group (if more than 1 Group listed in the byline) and/or Subgroup (eg, Steering Committee)
Kun	Wang			Guangdong Cancer Hospital	Guangzhou, Guangdong, China	principal investigator	
Yongmei	Yin			Jiangsu Province Hospital	Nanjing, Jiangsu, China	principal investigator	
Xiaoming	Zha			Jiangsu Province Hospital	Nanjing, Jiangsu, China	principal investigator	
Haiqing	Zhang			Central Hospital of Dalian	Dalian, Liaoning, China	principal investigator	
