# Supplementary material for: Trends in Disparities and Transitions of Treatment in Patients With Early Breast Cancer in China and the US, 2011 to 2021
Source: JAMA Netw Open. 2023 Jun 30;6(6):e2321388. doi: 10.1001/jamanetworkopen.2023.21388 (PMC10314317; doi:10.1001/jamanetworkopen.2023.21388)
Supplement: Supplement 3. — Data Sharing Statement [file jamanetwopen-e2321388-s003.pdf]

Data Sharing Statement

Li. Trends in Disparities and Transitions of Treatment in Patients With Early Breast Cancer in China and the US, 2011 to 2021. *JAMA Netw Open*. Published June 30, 2023. doi:10.1001/jamanetworkopen.2023.21388

Data
Data available: Yes
Data types: Deidentified participant data
How to access data: Emails should be sent to the address below to obtain the shared data from CSCO BC database(csco_bc@csco.org.cn) and that from Flatiron database (1020202613@cpu.edu.cn)
When available: With publication

Supporting Documents
Document types: None

Additional Information
Who can access the data: Data will be made available to these researchers whose proposed use of the data has been approved. Emails should be sent to the address below to obtain the shared data from CSCO BC database(csco_bc@csco.org.cn) and that from Flatiron database (1020202613@cpu.edu.cn)
Types of analyses: We may balance the potential benefits and risks for each request and then provide the data that could be shared.
Mechanisms of data availability: with investigator support
